# Supplementary material for: Leucine Contributes to Copper Stress Tolerance in Peach (Prunus persica) Seedlings by Enhancing Photosynthesis and the Antioxidant Defense System
Source: Antioxidants (Basel). 2022 Dec 13;11(12):2455. doi: 10.3390/antiox11122455 (PMC9774504; doi:10.3390/antiox11122455)
Supplement: Supplementary file 1 [file antioxidants-11-02455-s001.zip › antioxidants-2056822-supplementary.pdf]

Table S1. Primer sequences.

Gene	Primer name	Primer sequence
PpSOD(*LOC18775015*)	SOD -F	AGCCCACACTTTGATGCTCT
	SOD -R	AGGAGTGAGGAAGTGGGTGA
PpPOD(*LOC18788700*)	POD -F	ATCAGGGGCTCACACAATCG
	POD -R	ACAGGTGGGCATGTGGATTT
PpCAT(*LOC18777304*)	CAT -F	AGGGATCCTCGTGGTTTTGC
	CAT -R	TTCGGTTTGAGAGCATGGAC
PpGPx(ppa010584m)	GPx - F	AAATCTTCACTTGGGTCATCTAAATC
	GPx - R	CTCTGTAGCTGCTCTTGCATAAACAC
PpeCAT(ppa004776m)	CAT - F	CACA TGGAAGGCTCTGGTGT
	CAT - R	CCTTGGTAGCA TGGCTGTGA
PpDHAR(ppa011390m)	DHAR - F	TGATGTTCTTGGCGACTG
	DHAR - R	CTCTGGATTCACTTCGGTAA
PpActin(*LOC18779708*)	Actin-F	GTTATTCTTCATCGGCGTCTTCG
	Actin-R	CTTCACCATTCCAGTTCCATTGTC

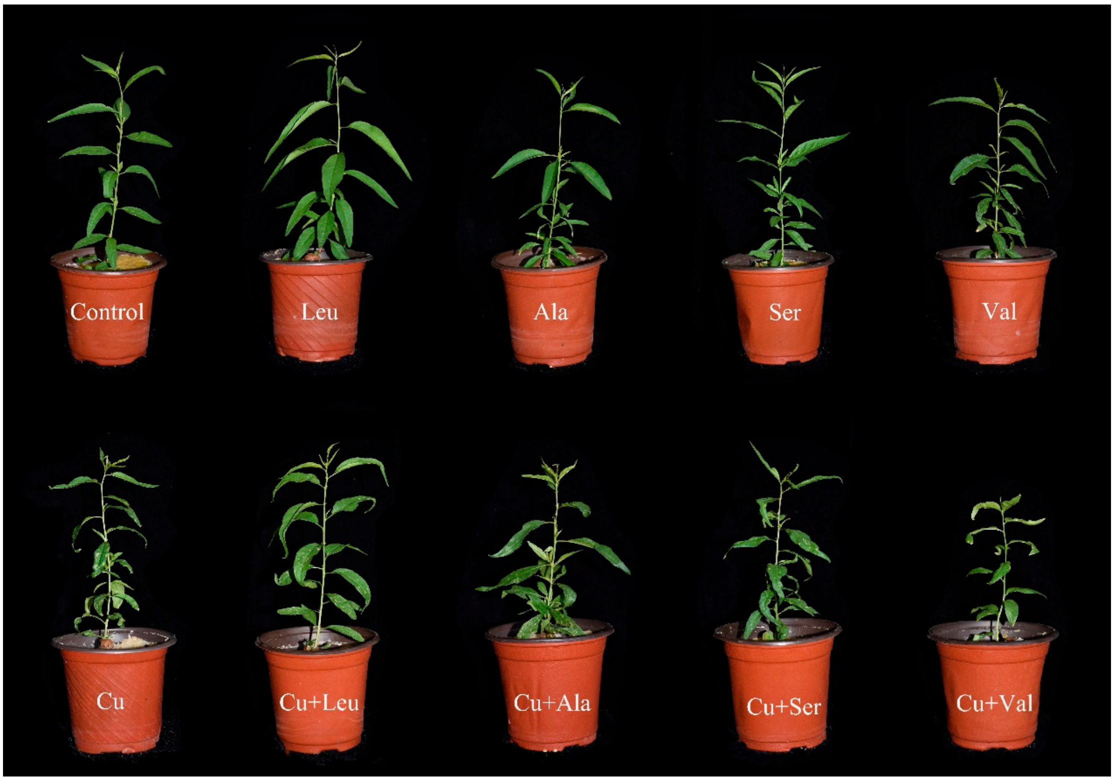

Figure S1. growth of 66-day-old peach seedlings under different amino acid treatments.

In the pre-experiment, peach seedlings under copper stress were treated with three concentrations of amino acids: 1 mmol · L^{-1}, 10 mmol · L^{-1} and 20 mmol · L^{-1}, respectively. It was found that leucine at the concentration of 10 mmol · L^{-1} was the most effective for reducing Cu stress in peach seedlings. Figure. S1 shows that Leu, Ala (alanine), Ser (serine) and Val (valine) have different effects on the growth and development of peach seedlings under normal conditions, and the application of Leu has the most obvious promotion effect on the growth of peach seedlings. Seedling development was blocked under Cu stress, and leaves withered and wilted. The application of Leu, Ala and Ser could alleviate the damage of peach seedlings under Cu stress, among which Leu had the most obvious effect. Therefore, we selected four treatments, Control, Leu, Cu and Cu+Leu, to explore the mechanism of Leu alleviating Cu stress.

Table S2. Effect of exogenous leucine on root architecture of peach seedlings under copper stress.

Treatment	Length (cm)	Surface area (cm^2)	Average diameter (mm)	Volume (cm^3)	Root tips	Forks
Control	246.7±12.7a	38.5±2.8a	0.50±0.02b	0.53±0.02a	644.8±23.2a	1081.0±52.9a
Leu	253.2±14.2a	41.52±3.2a	0.52+0.05b	0.53±0.03a	653.7±32.1a	1053.3±45.6a
Cu	107.1±1.7c	17.3±0.3c	0.63±0.02a	0.24±0.01c	167.4±6.0c	298.9±20.6c
Cu + Leu	134.8±5.0b	28.9±1.4b	0.51±0.03b	0.43±0.01b	268.9±8.1b	829.2±38.3b

Exogenous leucine promoted root growth of peach seedlings under copper stress. Further analysis of the root architecture showed that under copper stress, the total length, area, and volume of roots, number of root tips, and bifurcation of roots significantly reduced while the average root diameter significantly increased (Table .S2). The number of fine roots also decreased under copper stress (Figure. 5a). However, the total length, area, and volume of roots, number of root tips, and bifurcation of roots increased by 33.1%, 12.0%, 21.4%, 42.4%, and 57.3%, respectively, and the average diameter of roots decreased by 15.5% with exogenous leucine compared with those under copper stress alone (Table .S2). These results indicate that exogenous leucine promoted fine roots in peach seedlings under copper stress.

Table S3. Effect of exogenous leucine on the copper ions content of peach seedlings under copper stress.

Treatment	Leaves (mg/kg)	Stems (mg/kg)	Roots (mg/kg)
Control	7.10±0.18c	4.69±0.25c	23.70±0.29c
Leu	7.15±0.21c	4.53±0.31c	22.59±0.31c
Cu	64.82±4.14a	35.72±5.23a	213.22±25.20a
Cu + Leu	34.95±1.71b	25.06±1.19b	138.48±8.53b

Datas are presented as mean ± SD (n = 3). Different lowercase letters indicate significant differences among different treatments (Duncan test, $p < 0.05$).

The copper ions contents in the leaves, stems, and roots of peach seedlings under copper stress were significantly higher than (9.1, 7.7 and 9.0 times, respectively) those in the control

seedlings (Table .S3). However, the contents in leaves, stems, and roots of seedlings decreased significantly by 46.06%, 29.84% and 35.08%, respectively, compared with those in the seedlings under copper stress alone.
